# The Identification of Mutation in *BMP15* Gene Associated with Litter Size in Xinjiang Cele Black Sheep

**DOI:** 10.3390/ani11030668

**Published:** 2021-03-03

**Authors:** Zhi-gang Niu, Jin Qin, Yao Jiang, Xiang-Dong Ding, Yu-gong Ding, Sen Tang, Hong-cai Shi

**Affiliations:** 1 Key Lab of Reproduction & Breeding Biotechnology of Grass Feeding Livestock of MOA, Xinjiang Academy of Animal Science, Urumqi 830000, China; xjnzg@126.com (Z.-g.N.); skdown@foxmail.com (Y.-g.D.); tangsen@xjaas.net (S.T.); 2 College of Animal Science and Technology, China Agricultural University, Beijing 100193, China; qinjin19921026@sina.com (J.Q.); jiangyao133996@126.com (Y.J.); xding@cau.edu.cn (X.-D.D.)

**Keywords:** Xinjiang Cele black sheep, bone morphogenetic protein 15 gene, mutation, fecundity, litter size

## Abstract

**Simple Summary:**

The gene for Bone Morphogenetic Protein 15 has multiple single-nucleotide polymorphism sites related to fertility in sheep. To increase fertility, a better understanding of the regulation of Bone Morphogenetic Protein 15 in sheep is essential. This work describes the phenotype and molecular characteristics of a mutation identified in the Bone Morphogenetic Protein 15 gene of Cele black sheep in Xinjiang, China. This mutation may affect the fertility of sheep, which is very useful for breeding purposes.

**Abstract:**

The Bone Morphogenetic Protein 15 (*BMP15*) gene is known to have multiple single-nucleotide polymorphism sites associated with sheep fecundity. This study used gene sequence analysis and mutation detection assays for *BMP15* by using 205 blood samples of ewes with known lambing records. Sequence analysis showed that mutation B1 missed the CTT base in exon 1 at positions 28–30, leading to a leucine deletion in the BMP15 protein. Litter size of ewes differed significantly between BB and B+ genotypes of B1 (*p* < 0.05); however, the differences between wild genotype (++) and homozygous (BB) or wild genotype (++) and heterozygous (B+) were not significant (*p* > 0.05). Another mutation, T755C, is a T-to-C base change at position 755 of exon 2, resulting in leucine replacement by proline at this position of the BMP15 protein (p.L252P). Two genotypes were identified in the flock: heterozygous (E+) and wild-type genotype (++). Ewes with heterozygous (E+) p.L252P had significantly larger litter sizes than those with the wild-type genotype (*p* < 0.05). Comprehensive analysis suggests that p.L252P is a mutation that affects fecundity in Cele black sheep.

## 1. Introduction

Cele black sheep are a prolific sheep breed in Xinjiang, China. This breed produces more lambs per litter and has more estrous cycles within a year than other breeds in the same area. Although the environment of Xinjiang is very harsh, with hot summers, cold winters, and windy as well as dry conditions all year round, ewes of this breed produce 2–4 lambs per litter.

In recent years, substantial progress has been made in the understanding of major genes for the prolificacy of sheep throughout the world. A number of studies showed that major genes offer the potential to significantly increase the reproductive performance of sheep flocks [1,2,3]. The following three classes of fecundity genes (*Fec*) have been identified in sheep: bone morphogenetic protein receptor type IB (*BMPR-IB*), known as FecB on chromosome 6, growth differentiation factor 9 (*GDF9*), known as FecG on chromosome 5, and Bone Morphogenetic Protein 15 (*BMP15*), known as *FecX* on the X chromosome. Interestingly, all three *Fec* genes belong to the *TGF-β* super family [4,5,6].

We previously identified mutations in FecB of the *BMPR-IB* gene associated with increased litter sizes in Cele black sheep. However, the litter sizes of wild-type genotypes (++) of the BMPR-IB gene were higher than (−/+) and (−/−), reaching 162%. Other genes must, therefore, also control ovulation in Cele black sheep [7].

The expression of the *BMP15* gene was confirmed in the sheep ovary by RT-PCR. In situ hybridization showed that *BMP15* mRNA in the sheep ovary is only expressed in oocytes, and its encoded product plays an important role in oocyte development [8]. The BMP15 gene is located in chromosome X of sheep and consists of two exons encoded by 1179 nucleotides. This gene encodes a prepropeptide of 393 amino acid residues. Active mature *BMP15* peptide has a length of 125 amino acids.

At present, multiple mutations related to the fecundity of the *BMP15* gene have been identified in sheep. Ewes of mutant heterozygous individuals achieved a higher ovulation number, while homozygous mutant individuals were completely infertile because of the absence of primary follicles in the ovary [8,9,10,11] (e.g., *FecXI*, *FecXB*, *FecXL*, *FecXH*, *FecXG*, and *FecXR*). Galloway identified two different independent point mutations in BMP15: Inverdale (FecXL) and Hanna (FecXH) [8]. Cambridge and Belclare sheep have the FecXG for *BMP15* (B2, g. C718T) and FecXB for *BMP15* (B4, g. G1100T), and mutations B1 (CTT missed) and B3 (g. T747C) were also identified [11]. FecXI and FecXH change the amino residue of the mature *BMP15* protein, thus changing the function of *BMP15* and increasing ovulation of ewes [8]. Mutations in the *BMP15* gene have also been reported in Lacaune sheep [12]. This mutation leads to five naturally occurring mutations in the *BMP15* gene that have been associated with the modulation of ovarian function in Lacaune sheep. Indeed, FecXL are similar to other FecX mutations, which have been associated with increased ovulation rates or sterility. A 17-bp deletion in *BMP15* has been identified in Rasa Aragonesa sheep of Spain [13,14]. Exon 2 site 525–541 of the *BMP15* gene was missing 17 nucleotides and was named FecXR. In contrast, homozygous ewes with two mutations of FecXGr and FecX0 are hyperprolific [15]. A novel mutation Fex Bar of *BMP15* exhibited streaky ovaries with a blockade at the primary stage of folliculogenesis, as shown by histochemistry [2].

This study firstly verified the polymorphism of the four sites of FecXI, FecXH, FecXG, and FecXB of the *BMP15* gene in a population of Cele black sheep. Furthermore, other mutations in the *BMP15* gene were explored and their effects on litter size were tested. This work describes the phenotypic and molecular characterization of a mutation identified in the *BMP15* gene in Cele black sheep.

## 2. Materials and Methods

### 2.1. Experimental Animals

A total of 205 healthy Cele black sheep ewes, 1–2 years old, were chosen in Cele County, Xinjiang Province, China. All of these Cele black sheep ewes were lambing second borns and lambing in the spring. Briefly, 205 blood samples were collected from the jugular vein directly into tubes containing heparin sodium. Samples were then transferred to the laboratory freezer (−20 °C). At the start of the breeding season, ewes were randomly mated with rams. The ewes were directly obtained from farmers and nearby villages of Cele County during 2008–2010 and were not treated with hormones to synchronize estrus.

### 2.2. DNA Extraction

DNA was extracted from blood using a slightly modified standard phenol chloroform procedure [16]. DNA concentrations were assessed using a BioPhotometer plus (Eppendorf, Hamburg, Germany) and stored at −20 °C before use.

### 2.3. Detection of FecXI, FecXH, FecXG, and FecXB Mutation Sites of the BMP15 Gene

With reference to previous research [6,8,10], four pairs of primers were designed for FecXI, FecXH, FecXG, and FecXB to detect the *BMP15* gene. These primers are listed in Table 1.

The PCR reaction volume of 20 µL contained Taq DNA polymerase 1.5 U, 10 × buffer 2 µL, MgCl2 (25 mmol/L) 1.2 µL, dNTP (2.5 mmol/L) 2 µL, genomic DNA ~50 ng, as well as upstream and downstream primers (each 10 pM). Water was added at 20 µL.

The above sites were detected using the Touch-Down PCR reaction program. The reaction conditions were as follows: (1) 95 °C pre-denaturation for 5 min; (2) Annealing temperature for PCR was performed by gradient PCR which 95 °C denaturation for 30 s, 65 °C–51 °C (65 °C, 63 °C, 61 °C, 59 °C, 57 °C, 55 °C, 53 °C, 51 °C) annealing for 40 s, 72 °C extension for 30 s (each annealing temperature of 65 °C–53 °C for two cycles respectively and 51 °C for 19 cycles); (3) finally, extension at 72 °C for 5 min. The product was detected by 2% agarose gel electrophoresis.

Restriction enzyme digestion of the PCR product *BMP15* gene FecXH mutation (CT) was amplified with the Spe I enzyme. The *BMP15* gene FecXI mutation (TA) was amplified via Xba I endonuclease digestion. The *BMP15* gene FecXG mutation amplification product was digested with the Hinf I enzyme. The FecXB mutation was amplified and digested with Dde I endonuclease. The digestion reaction system used 4 μL PCR product, 1 μL 10× buffer, and 5 U endonuclease filled to 10 μL with double-distilled water. The digestion used a water bath at 37 °C for 12 h. The digested product was detected by 3% agarose electrophoresis.

### 2.4. Exons 1 and 2 of BMP15 Gene Sequence Analysis

The sheep *BMP15* genes were amplified using PCR with primers designed by Oligo version 6.0, using gene sequences published on GenBank (sheep genomic *BMP15* exon 1, AF236078; sheep genomic *BMP15* exon 2, AF236079).

The following PCR primers were used:

*BMP15* exon 1:

Forward 5′-TTTCATTTTTCCTTGCCCTATCC-3′;

Reverse 5′CCTGACAGAAAACTGACAGATCC-3′.

*BMP15* exon 2:

Forward 5′-TTCTCTGAGCTTCAGTTTCCTCG-3′;

Reverse 5′-TGCACCTTTGCCGTCACCTGCAT-3′.

Amplification was conducted for 35 cycles in a 20-μL reaction mixture, using 50 ng of DNA templates, 50 mm KCl, 10 mm Tris-HCL, 1.5 mm MgCl_2_, 200 μm of each dNTP, 5% DMSO, 10 pM of each primer, and 1.5 U of Taq DNA polymerase. Touchdown amplification was conducted using the following program: 95 °C for 5 min, followed by a gradient over 16 cycles of 95 °C for 45 s, 65 °C annealing for 60 s, and 72 °C extension for 90 s. The annealing temperature was subsequently decreased by 2 °C every second cycle; 19 cycles at 94 °C for 45 s, at 51 °C for 60 s, and at 72 °C for 90 s. The final extension at 72 °C lasted for 6 min. The PCR products were separated by electrophoresis at 85 V for 45 min in 1.5% agarose gels, and the results were visualized on a VDS system (UV and video).

Twelve samples from the flock were amplified; the size of *BMP15* exon 1 was 550 bp, and that of *BMP15* exon 2 was 1116 bp. The resulting PCR products were sequenced on an ABI 373 sequencer (Shanghai Biological Engineering Co., Ltd., Shanghai, China).

Two mutations were identified in the *BMP15* gene. In exon 1, the 28–30 CTT base was missing, which resulted in a predicted leucine deletion in *BMP15* protein (B1). At position 755 in exon 2, one T was changed to C, resulting in a predicted change from leucine to proline in *BMP15* protein (T755C).

### 2.5. Mutation Detection Assays

The sequences of forward and reverse primers for the amplification of the fragment that includes new mutants of *BMP15* genes are listed in Table 2. PCR was performed in 20 μL reaction mixture with 50 ng template DNA, 50 mM KCl, 10 mm Tris-HCL, 1.5 mm MgCl_2_, 200 μm of each dNTP, 5% DMSO, 10 pM of each primer, and 1.5 U of Taq DNA polymerase.

Tetra-primer amplification refractory polymorphism system PCR (Tetra-ARMS-PCR) was used to amplify B1 to identify the missing CTT using the four primers listed in Table 1. PCR products were separated on a 3% agarose gel and visualized with the VDS system. The fragment size showed fragments of 189 and 286 bp that were missing CTT (homozygous); 134, 189, and 286 bp (heterozygous); 134 and 286 bp that were the wild-type genotype (no missing CTT), and a fragment of 286 bp indicated the control size in each array.

To design the exon 2 forward primer, A was changed into G at the second end of the 3′ cleavage site to enhance recognition by the restriction enzyme Apa I. A total of 4 μL of PCR products was digested with 10 U Apa I, 1 μL 10× buffer, and ultra-pure water was added to a 10-μL final volume. This mixture was incubated at 37 °C for at least 12 h. The digested products were separated in an 8% polyacrylamide gel (29:1) for 8 h at 120 V. The gels were stained with silver nitrate and then observed in the VDS system. The homozygous sizes were 120 and 20 bp, heterozygous sizes were 140, 120, and 20 bp, and wild-type genotype (no mutation) size was 140 bp.

### 2.6. Statistical Analysis

Analysis of the association between genotypes and litter size was conducted by applying a general linear model (GLM) procedure using SAS software version 9.0 (SAS Institute Inc.). The model was described as:

Y = μ + a + e

where Y represents the value of litter size, μ represents the overall mean, a represents the effect of the genotype, and e represents the random residual error.

## 3. Results

### 3.1. Detection of FecXI, FecXH, FecXG, and FecXB Mutation Sites of the BMP15 Gene

Genomic DNA was PCR-amplified with primers at the FecXH site to obtain a target band with a size of 240 bp. After digestion with Spe I enzyme, it was detected by 8% non-denaturing PAGE electrophoresis. The results showed that the PCR product was not cleaved by the enzyme, and its size still remained at 240 bp (see Supplementary Figure S1A,B).

PCR amplification of genomic DNA with FecXI primers resulted in a target band with a size of 150 bp, and primer dimer bands also formed. However, this did not affect the enzymatic digestion. After Xba1 digestion, detection was performed by 8% non-denaturing PAGE electrophoresis, which identified multiple bands; however, their size was not the target band. The PCR product could not be cleaved by the enzyme. The type still retained its size of 150 bp, indicating a lack of polymorphism at this site in Cele black sheep (see Supplementary Figure S2A,B).

Genomic DNA was PCR-amplified with FecXG primers to obtain a 141-bp target band. After digestion with Hinf I endonuclease, the digested product was detected by 3% agarose electrophoresis. The result was only a 111-bp band, indicating that there was no polymorphism at this site in Cele black sheep (see Supplementary Figure S3A,B).

PCR amplification of genomic DNA with FecXB primers identified a target band of 153 bp. Although primer dimers were also found, this did not affect endonuclease digestion. After digestion, only a 122-bp band remained, indicating that there was no polymorphism at this site in Cele black sheep (see Supplementary Figure S4A,B).

### 3.2. Sequencing Results of the BMP15 Gene Coding Region

#### 3.2.1. Exon 1 of the BMP15 Gene Sequence

**Figure 1:**
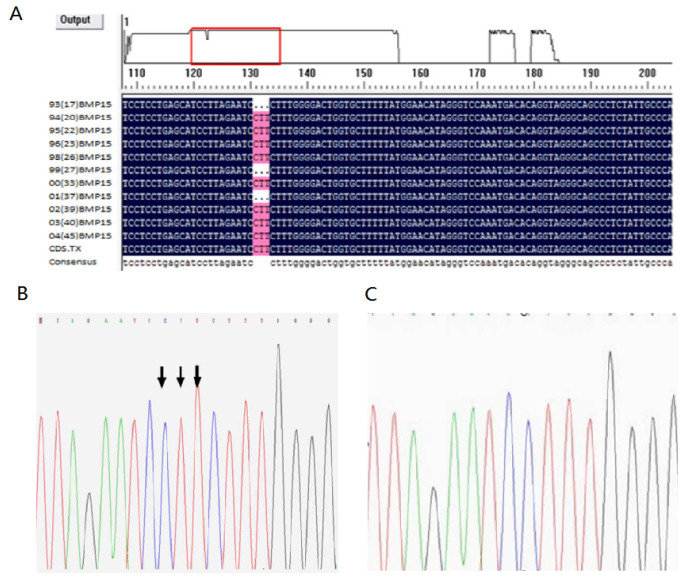
Sequencing results of exon 1 of the *BMP15* gene. (**A**) Comparison of sequence exon 1; (**B**) sequence of wild-type (without mutation) at E1+28-30 site of exon 1; (**C**) sequence of mutation (missing CTT) at E1+28-30 site of exon 1.

From each of the 12 samples, exon 1 of the *BMP15* gene was amplified and sequenced by an automated ABI DNA sequencer (model 373, PE Applied Biosystems). Comparison and blast data showed that the E1 site had a CTT deletion at 28–30 (Figure 1A–C).

#### 3.2.2. Exon 2 of the BMP15 Gene Sequence

**Figure 2:**
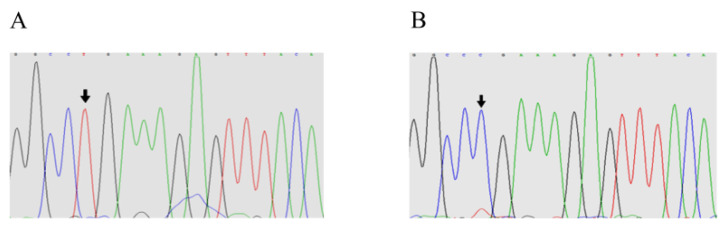
Sequencing results of exon 2 of the *BMP15* gene. (**A**) Sequence of wild-type E2+755 at the site of *BMP15* exon 2; (**B**) sequence of mutation (T–C) at the E2+755 site of *BMP15*.

Exon 2 of the *BMP15* PCR products was sequenced for each of the 12 samples. Position 755 of exon 2 was changed, and T was replaced by C. This was predicted to result in a change from leucine to proline (Figure 2A,B).

### 3.3. Mutation of the BMP15 Gene Polymorphism

**Figure 3:**
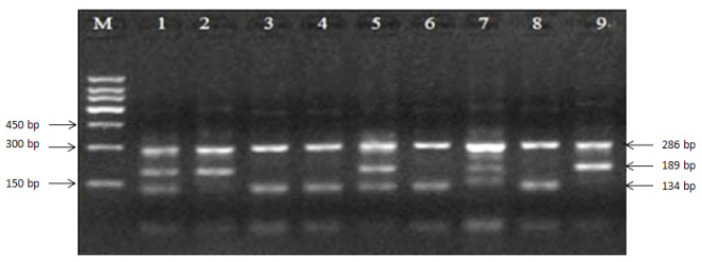
Tetra-primer amplification refractory polymorphism system PCR (Tetra-ARMS-PCR) products of E1+28-30 site of exon 1 in BMP15. M: 150-bp marker; wild-type genotype (++): 3, 4, 6, and 8; heterozygous (B+): 1, 5, and 7; homozygous (BB): 2 and 9.

A total of 205 ewes with reproduction records were tested using Tetra-ARMS-PCR methods for site E1+28-30. The 3% agarose gel showed that heterozygous (B+) samples for the mutation had fragments of all three sizes (134, 189, and 286 bp), homozygous (BB) had two sizes (189 and 286 bp), and the wild-type genotype (++) also had two sizes (134 and 286 bp, Figure 3).

**Figure 4:**
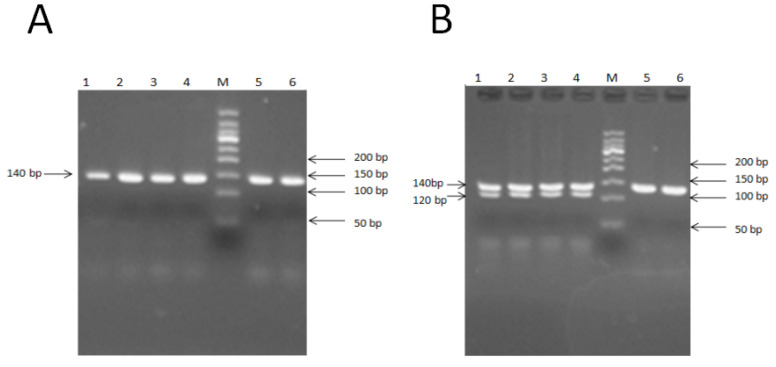
Mutation in BMP15 gene exon 2. (**A**) PCR products of exon 2, E2+755 site of BMP15. M: 50-bp marker; 1–6: 140-bp M and 150-bp marker; wild-type genotype (++): 3, 4, 6, and 8; heterozygous genotype (B+): 1, 5, and 7; homozygous genotype (BB): 2 and 9. (**B**) PCR-RFLP 8% polyacrylamide gel result of exon 2, E2+755 site of BMP15. M: 100-bp marker; wild-type genotype (++): 5 and 6; heterozygous genotype (B+): 1, 2, 3 and 4.

PCR-RFLP methods were used to identify E2+755 sites. PCR was conducted using primers with single mismatches to generate products containing restriction enzyme sites. The mismatch that was created in the appropriate primer to create the restriction enzyme Apa1 cleavage site was mentioned above (also see Figure 4A). The digested fragments were separated on an 8% polyacrylamide gel and were visualized with the VDS system. The fragments for the homozygous genotype (EE) were 120 and 20 bp; for the heterozygous genotype (E+), these were 140, 120, and 20 bp, and for the wild-type genotype (++), this was 140 bp, as the 20 bp was too small and moved out of the gel (Figure 4B).

### 3.4. Genotypic and Allelic Frequencies of the Mutation of the BMP15 Gene in Cele Black Sheep

The main genotypes in the investigated flock are the wild-type genotypes of E1+28-30 and E2+755 sites (Table 3). Both X^2^ values (*p* < 0.01) suggest that these genotypes significantly departed from the Hardy–Weinberg equilibrium, indicating that the flock was influenced by breeding or sampling.

### 3.5. Effect of Genotypes on Litter Size

The results of litter sizes of different genotypes are shown in Table 4. Both the heterozygous genotypes of E1+28-30 and E2+755 had larger litter sizes (2.15 and 2.20). The litter size of E1+28-30 wild-type (++) ewes was higher than that of homozygous (BB) ewes. For E2+755, only heterozygous (E+) and wild-type (++) genotypes were identified, and heterozygous (E+) ewes produced 0.33 more offspring on average than wild-type (++) ewes (*p* < 0.05).

## 4. Discussion

In mice, *BMP15* is specifically expressed in the oocyte, initially at the one-layer primary follicle stage, which continues through ovulation. Interestingly, *BMP15* is most closely related to and shares a coincident expression pattern with the mouse GDF9 gene, which is essential for female fertility [17]. In mammals with a low ovulation rate phenotype, both oocyte-derived GDF9 and *BMP15* proteins are essential for normal follicular development, both at the early and later stages of oocyte growth. Regulation of *BMP15*, GDF9, or both is potentially a new technique to enhance fecundity in mammals [18,19]. In ewes immunized with different *BMP15* or GDF9 peptide sequences, antibodies generated against the N-terminal region of *BMP15* or GDF9 were potent inhibitors of the paracrine actions of these oocyte-secreted factors both in vivo and in vitro. This induced an ovulation phenotype that blocks the biological actions of *BMP15* or GDF9 at their N-termini, suggesting potential as contraceptives or sterilizing agents [20]. Similarly, in cattle, immunization against *BMP15* and/or GDF9 altered follicle development [21], and *BMP15* appears to be important in promoting follicle growth at the early stages while restraining the number of dominant preovulatory follicles. *BMP15* is a key determinant of both ovulation number and litter size in sheep and cattle [22].

Mutations of the *BMP15* gene have been identified in several sheep breeds across the world, indicating that the heterozygous genotype (B+) influences the additive effect on ovulation rate. Apparently, recombination or mutations are frequent within the *BMP15* gene in sheep. FecXI, FecXH, FecXG, and FecXB of the *BMP15* gene are closely related to the fetal sex of several sheep. No polymorphisms were found in the investigated population of Cele black sheep, indicating that these are not associated with the number of high-yielding lambs in this breed.

Dube discovered two mutations in the *BMP15* gene, both of which result in high yields of Inverdale sheep [17]. In their report, the Inverdale (FecXI) allele or the Hanna (FecXH) allele increased litter size, while homozygous ewes that inherited alleles from both parents had small, undeveloped ovaries and were infertile. B1 mutation has also been identified at the 28-30 position of exon 1 with a three-base deletion of CTT, which leads to *BMP15* protein deletion of leucine. This locus has previously been shown to be polymorphic (without any phenotypic effect) in the predicted signal sequence, while a subset of sheep have two leucine codons (CTT) at this position and other sheep have only one. The present study also found B1 deletion, and three genotypes were detected. Analysis showed that the homozygous genotype (BB) had the smallest litter size compared with the heterozygous genotype (B+) with the highest litter size and the wild-type genotype (++). The differences between homozygous (BB) and wild-type (++) phenotypes were not significant (*p* > 0.05) and neither were the differences between heterozygous (B+) and wild-type (++) phenotypes (*p* > 0.05). Furthermore, two leucine codons were detected in Cele black sheep. These results suggest that B1 deletion does not increase ovulation in Cele black sheep.

The presented results show that *BMP15* has a mutation at position 755 of exon 2 in Cele black sheep, which results in proline instead of leucine formation (p.L252P). Only heterozygous (E+) and wild-type (++) genotypes were detected in the 205 tested ewes. No homozygous genotype (EE) was observed. A possible reason is that only the mutations in ewes with offspring were tested, and the allele frequency of mutation E was low (0.102). This may have resulted in a particularly low frequency of the homozygous genotype (EE) or EE may be sterile. Consequently, ewes with offspring records were unavailable, and more samples are needed in the future. This study showed that heterozygous (E+) ewes have larger litter sizes than wild-type (++) ewes (*p* < 0.05), suggesting that this mutation may have influenced the litter size by a change in the structure and function of the BMP15 protein.

## 5. Conclusions

According to many studies of the *BMP15* gene in sheep, three characteristics of changes in gene function can be summarized: (1) Heterozygous mutations of *BMP15* increase the ovulation rate and ewes have more progeny; (2) homozygous individuals show abnormal ovarian development, rudimentary “streak” ovaries, and sterility; (3) all mutations are within the *BMP15* gene coding region, which disrupts the *BMP15* protein and causes either premature termination or produces an amino acid change in the mature coding region, which changes the protein function. These studies support that p.L252P is associated with reproductive effects in Cele black sheep. In this study, no EE genotype of p.L252P was observed. To date, it is not clear if ewes with the EE genotype are sterile or if the sample size was insufficient. In summary, this locus is a major gene which controls the multi-fetal performance of Cele black sheep.

## Figures and Tables

**Table 1:** Primers of FecXI, FecXH, FecXG, and FecXB mutation sites of the Bone Morphogenetic Protein 15 (*BMP15)* gene.

Site	Primer Sequence	Size
FecXG	Forward 5′-CACTGTCTTCTTGTTACTGTATTTCAATGAGAC-3′ Reverse 5′-GATGCAATACTGCCTGCTTG-3′	141 bp
FecXB	Forward 5′-GCCTTCCTGTGTCCCTTATAAGTATGTTCCCCTTA-3′ Reverse 5′-TACTTTCAGGCCCATCATGCTCC-3′	153 bp
FecXI	Forward 5′-GAAAGTAACCAGTGTTCCCTCCACCCTTTTCT-3′ Reverse 5′-CATGATTGGGAGAATTGAGACC-3′	150 bp
FecXH	Forward 5′-TATTTCAATGACACTCAGAG-3′ Reverse 5′-GAGCAATGATCCAGTGATCCCA-3′	240 bp

**Table 2:** Primers of mutational sites in *BMP15.*

Site	Primer Sequence	Size
E1+28-30	Forward 5′-TGTTACCCATGTAAAAGGAAAGG-3′ Reverse 5′-AAAAGCACCAGTCCCCAAAGAAG-3′ Forward 5′-TGTTACCCATGTAAAAGGAAAGG-3′ Reverse 5′-ACCGTAAGGGATGCCCTAAGACC-3′	134 bp 189 bp 286 bp
E2+755	Forward 5′-GAAGACCAAACCTCTCCCTAAGG-3′ Reverse 5′-TACTTTCAGGCCCATCATGCTCC-3′	140 bp

PCR cycles used the Touch-Down program as mentioned above.

**Table 3:** Genotypic and allelic frequencies of E1 = 28-30 and E2 = 755 sites in Cele black sheep.

Site	Ewe Number	X^2^	Genotypic Frequency (Number)			Allelic Frequency	
			++	B+/E+	BB/EE	B/E	+
E1+28-30	200	X^2^ = 13.699 (*p* = 0.001)	0.615 (123)	0.275 (55)	0.11 (22)	0.247	0.753
E2+755	205	X^2^ = 26.46 (*p* = 0.0001)	0.795 (163)	0.205 (42)	0	0.102	0.898

**Table 4:** Least squares mean and standard errors for litter sizes of different *BMP15* mutations.

Scheme	Ewe Number	Genotype	Litter Size
E1+28-30 (B1)	22	BB	1.64 ± 0.181 ^a^
	55	B+	2.15 ± 0.114 ^b^
	123	++	1.90 ± 0.077 ^ab^
E2+755	42	E+	2.20 ± 0.129 ^b^
	163	++	1.87 ± 0.066 ^a^

^a,b^ In each genotype group, these different subscript letters represent significant differences at a significance level of 0.05.

## Data Availability

The data in this study was mutation sites detect by using the restriction enzyme. No new data were created or analyzed in this study. Data sharing is not applicable to this article.

## Supplementary Materials

The following materials are available online at https://www.mdpi.com/2076-2615/11/3/668/s1, Figure S1A: PCR products of the FecX^H^ gene; Figure S1B: Digestion of PCR products of the FecX^H^ gene. Figure S2A: PCR products of the FecX^I^ gene; Figure S2B: Digestion of PCR products of the FecX^I^ gene; Figure S3A: PCR products of the FecX^G^ gene; Figure S3B: Digestion of PCR products of the FecX^G^ gene; Figure S4A: PCR products of the FecX^B^ gene; Figure S4B: Digestion of PCR products of the Fec^B^ gene.
