# Supplementary figures and images for: The Identification of Mutation in BMP15 Gene Associated with Litter Size in Xinjiang Cele Black Sheep
Source: Animals (Basel). 2021 Mar 3;11(3):668. doi: 10.3390/ani11030668 (PMC8001854; doi:10.3390/ani11030668)

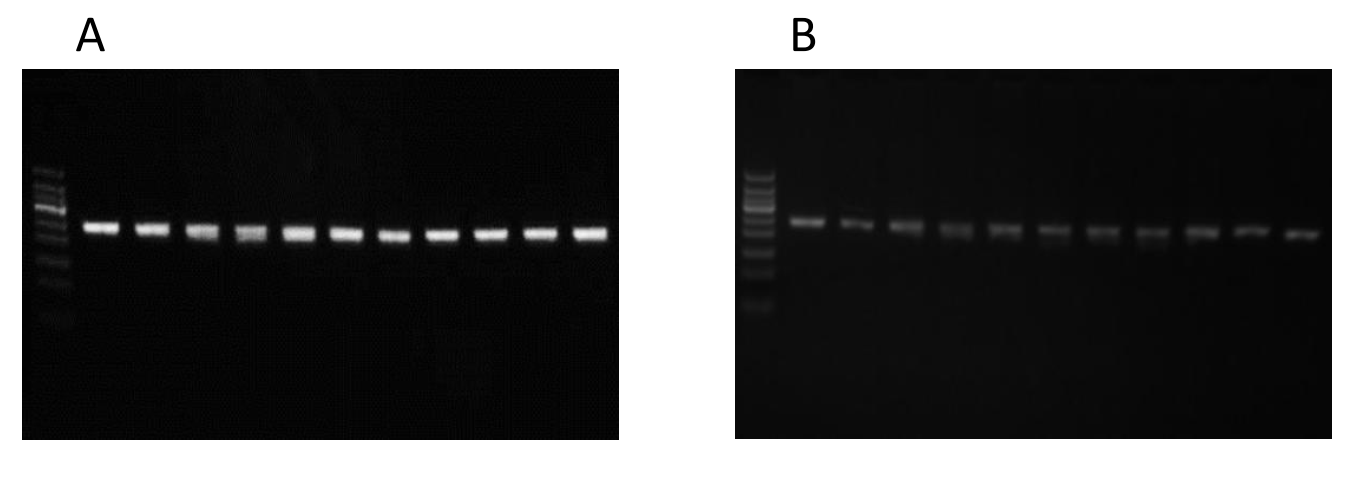

Supplement: Supplementary file 1 [file animals-11-00668-s001.zip › Supplementary 1.tif]

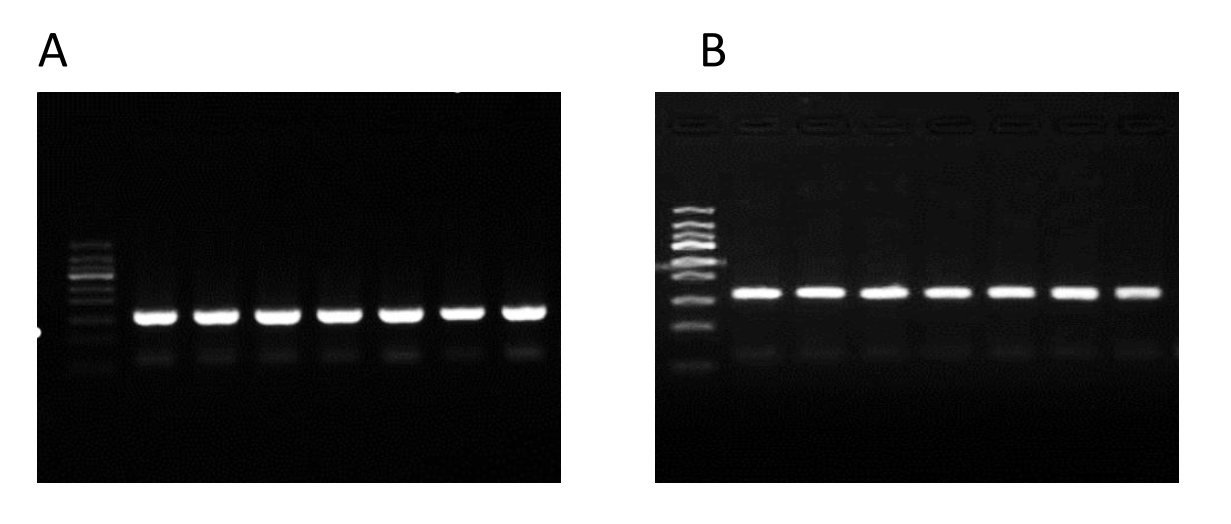

Supplement: Supplementary file 1 [file animals-11-00668-s001.zip › Supplementary 2.tif]

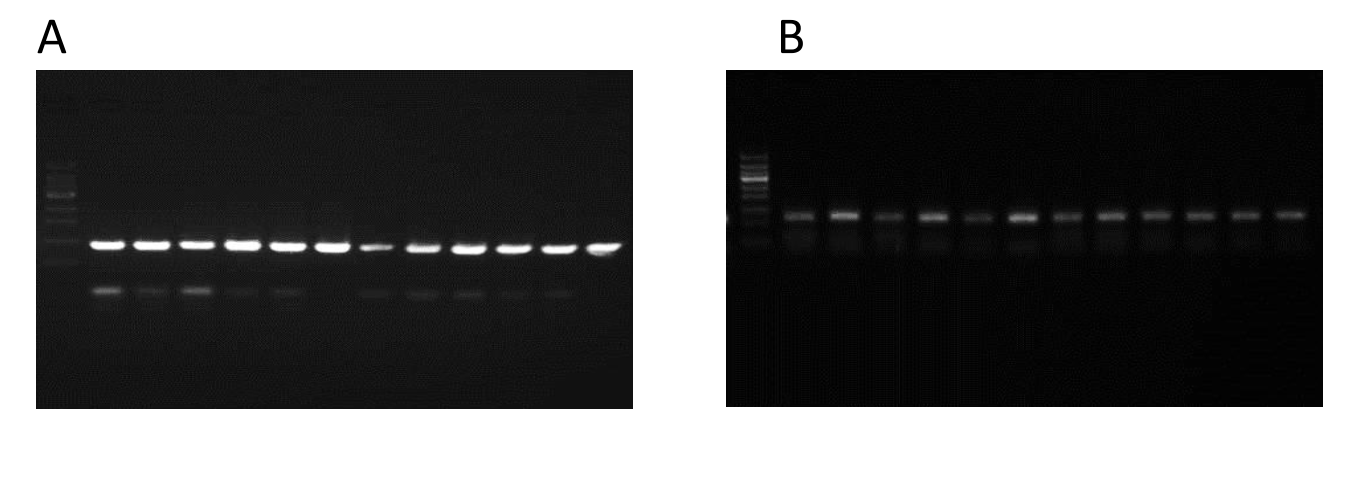

Supplement: Supplementary file 1 [file animals-11-00668-s001.zip › Supplementary 3.tif]

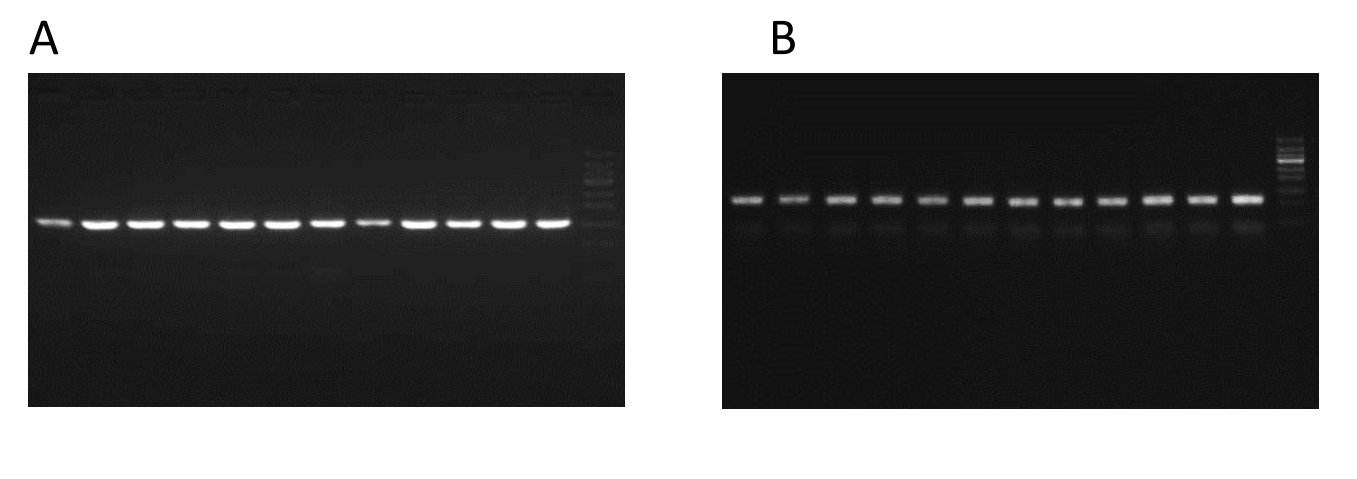

Supplement: Supplementary file 1 [file animals-11-00668-s001.zip › Supplementary 4.tif]
